# Affect and Intellect in Judgments: Factors Which Determine Level of Evaluative Heterogeneity

**DOI:** 10.3389/fpsyg.2016.00569

**Published:** 2016-04-25

**Authors:** Maria Jarymowicz

**Affiliations:** Faculty of Psychology, University of WarsawWarsaw, Poland

**Keywords:** extreme vs. moderate appraisals, implicit priming, prejudice, limits of affects' influence

What determines whether someone has a one-sided view of the world or perceives both the negative and positive aspects of something? To understand the factors which determine the extent to which human judgments are absolute we have to take into account studies of conscious and non-conscious information processing (Reber, 1993; Underwood, 1996; Hassin et al., 2005), and implicit and explicit emotions (Zajonc, 1980; Greenwald and Banaji, 1995; Ohme, 2007), which can be interpreted as evaluative processes of different types (Reykowski, 1968; Oatley and Jenkins, 1996; Sander and Scherer, 2009). Understanding human judgment requires a knowledge of psychology and neurobiology (Damasio, 1994; LeDoux, 1996, 2012; Sander et al., 2003; Sander and Scherer, 2009; Armony and Vuilleumier, 2013), both of which help to explain the conditions in which individuals make extreme, absolute evaluations and the mechanisms which enable more complex, moderate and nuanced appraisals.

We argue that models of judgment processes should refer to the dual mind theories (Epstein, 1990; Chaiken and Trope, 1999; Liberman, 2003; Deutsch and Strack, 2006; Kahneman, 2011), and emphasize the distinction between affective (automatic) vs. intellectual (reflective) premises of evaluative appraisals (Jarymowicz and Bar-Tal, 2006; Jarymowicz, 2009; Jarymowicz and Imbir, 2015).

## Affective vs. intellectual evaluations

Affect can be viewed as a direct, automatic response to a stimulus which “by-passes the will” (Gazzaniga, 2011) and influences cognition, evaluation, motivation and behavior (Zajonc, 1984; Bargh and Chartrand, 1999; Cacioppo and Gardner, 1999; Pessoa, 2008). Primary affective states (generated at subcortical level) are diffuse and lead to global, and often extreme, one-sided appraisals (Plutchik, 1980; Zajonc, 1980; Cacioppo and Berntson, 1994). Moreover, these judgments may be based on implicit, unacknowledged premises and associated with high subjective certainty (Balas et al., 2012). Unfortunately, such unquestioned judgments become rigid and resistant to change.

Intellect, the basis of deliberative thinking, enables individuals to reflect in more or less general terms on reality, the past and the anticipated future, and to develop and use evaluative concepts, including abstract concepts related to values (e.g., *equality, justice,* and *humanism*). Although such moral concepts are so complex that they are never well-defined, reflective cognition can result in the formation of moral standards, which are then used as points of reference for judgments about reality (Reykowski, 1989; Krzemionka, 1993; Baumeister et al., 2007). As an individual develops a capacity for deliberative thinking he or she acquires the ability to apply new types of evaluative criteria and verbalize judgments based on these criteria; this gives rise to secondary affective states (Zajonc, 1980; Rolls, 2000). Secondary affect does not dominate evaluative thinking processes. An individual is able to perceive both negative and positive elements of a given object and consequently his or her appraisal will be more nuanced, we will refer to such judgments as “heterogeneous” (e.g., one might be aware that out-group members have some negative as well as positive qualities).

## The concept of evaluative heterogeneity

We posit that the capacity for evaluations based on affect is universal, whilst the capacity for evaluations based on intellectual premises is less widespread. Judgments based on affect are effortless, whereas evaluations based on intellectual premises require conscious attention and deliberative thinking; in other words they require the evaluator to invest time, energy and cognitive capacity. It follows that there is considerable individual variation in the relative proportions of affect-based (automatic) and intellect-based (reflective) evaluations; we refer to an individual's disposition to base evaluations on intellectual premises as his or her “level of evaluative heterogeneity” (Jarymowicz, 2008, 2012). *Evaluative heterogeneity* is defined as the ability to make nuanced appraisals which take into account both the positive and negative attributes of an object.

Our studies were based on following assumptions: (A) intellectual/reflective evaluative activity results in a tendency to connect the evaluation process with a search for premises on which to base evaluations; (B) this tendency reduces the influence of irrelevant, affective stimuli on judgments; (C) this reduction in the influence of affective factors applies to both explicit and implicit evaluative processes.

Our hypothesis and empirical findings are described below. We expected to find a correlation between evaluative heterogeneity and resistance to the influence of irrelevant affective factors. The studies considered two correlates: (1) the independence of explicit appraisals on implicit affective priming and (2) the limits of automatic in-group favoritism.

## Measurement of evaluative heterogeneity

Measurement of evaluative heterogeneity was based on the assumption that this disposition will be reflected in an individual's spontaneous references to the negative as well as the positive attributes of a given object. In this context “spontaneous” means unprompted (e.g., by the experimenter); rather than having respondents choose attributes from a check list we required them to specify the attributes they associated with a given object by answering an open question.

We decided to use objects that are usually evaluated positively (to avoid overly strong interference from negative affect). In all the studies mentioned below participants were asked to list “good and bad aspects of patriotism” (patriotism is typically regarded as a very positive quality in Poland).

The “Dilemmas and Discussions” technique (Karwowska and Jarymowicz, 2003) involves presenting respondents with an introduction which points out that some social attitudes (e.g., to one's country, to migration, abortion, children adoption etc.) are controversial, and explains that “in this study we are asking young people what they think about patriotism.” Then respondents are presented with a table consisting of two columns entitled “Positive aspects of patriotism” and “Negative aspects of patriotism” and the instruction “Please list as many negative and positive aspects of patriotic attitudes as possible.” Level of evaluative heterogeneity is indexed as the proportion of a number of negative attributes to a sum of all generated attributes.

## The relationship between evaluative heterogeneity and resistance to the influence of subliminal affective stimuli

In series of studies we used divers versions of the subliminal affective priming paradigm (Murphy and Zajonc, 1993). In the classic version of the task participants are asked to give affective evaluations of unfamiliar, neutral signs (Chinese ideograms) using a scale ranging from “I like it” to :I don't like it.” All responses are primed by displaying a neutral or affective subliminal stimulus for several milliseconds before the sign is presented. The priming stimuli are photographs of human faces showing neutral, positive (e.g., joyful) or negative (e.g., disgusted) expressions. In version used by some other authors (Ohme, 2007) participants are presented with Chinese ideograms which are described as “symbols of human traits” and asked to make an intuitive judgment as to whether a given ideogram represents a positive or a negative trait. In our modification the participant had to decide the extent to which a given ideogram represents a trait characteristic of him or her (Błaszczak and Imbir, 2012). All the data showed that implicit stimuli affect explicit judgments and behavior; however in all the studies we observed inter-individual variation in the extent of this influence (Jarymowicz, 2008; Karwowska and Kobylińska, 2014).

Karwowska (2001) introduced an important modification to the affective priming impact index to distinguish between participants who were more and less resistant to the influence of priming. The modified index was based on the difference between explicit appraisals made after negative or positive priming (with faces expressing joy or disgust) and appraisals made under control conditions (priming with a neutral face). A small difference between appraisals made after affective and neutral priming indicates that explicit appraisals are relatively independent of the influence of affective priming.

In several studies level of evaluative heterogeneity was found to be associated with the relative influence of affective priming, i.e., in individuals with high evaluative heterogeneity there was a relatively small difference between the explicit appraisals of neutral objects made following affective and neutral priming (Jarymowicz, 2008). These data are consistent with the results of experimental studies in which we stimulated reflective evaluative thinking and then provoked reactions to the subliminal affective priming (Karwowska and Kobylińska, 2014). The data suggest that the reflective system may inhibit automatic reactions (Imbir and Jarymowicz, 2013).

## The relationship between evaluative heterogeneity and baseless in-group favoritism

In this section we discuss studies which suggest that evaluative heterogeneity is related to the extent to which social judgments are based on prejudices. We define a prejudice (Jarymowicz and Bar-Tal, 2006) as a pattern of direct, automatic affective reactions to an object which influences information processing and can lead to formation of rigid cognitive schemata, i.e., stereotypes. Links between primary affective states and cognitive processes—and hence links between prejudices and stereotypes—are usually very stable. We postulate that changes in these associations may be due to the development of reflective evaluative standards and hence new approaches to social perception and evaluation of the world (Jarymowicz and Imbir, 2015).

Several of our empirical studies investigated automatic in-group favoritism and out-group discrimination (Jarymowicz, 2006, 2008). These studies were based on the assumption that any sign of social belonging (including subliminal signs) can generate diffuse affect and thus lead to simplified, absolute, homogeneous evaluations. We expected that people with high evaluative heterogeneity would be less vulnerable to this kind of affective influence.

In our research on explicit attitudes we asked participants for their opinions on diverse social objects, varying how direct the method of assessing opinion was. In one study, for example, participants were asked questions about the achievements of Poland and various other non-European countries in several domains where achievement is difficult to evaluate (such as care for the elderly or provision for gifted children). Our Polish participants viewed Poland as more successful than other countries. In another study we asked questions about the rights of ethnic and sexual minorities. A notable finding was that in all the studies in-group favoritism and aversion to out-group members were relatively low among participants with relatively high level of evaluative heterogeneity (Jarymowicz, 2013).

## Final comment

Both the psychological and neurobiological data indicate importance of the distinction between affective/automatic and intellectual/reflective evaluative systems (Zajonc, 1980; LeDoux, 1996; Jarymowicz et al., 2013; Imbir et al., 2015, 2016; Jarymowicz and Imbir, 2015). Like other dual mind theories (Epstein, 1990; Deutsch and Strack, 2006; Kahneman, 2011), the concept of dual evaluative systems can explain why an individual's reactions may vary across time and circumstances. But this concept leads to question about possible interferences between the two systems; one might ask under what conditions each system dominates.

The explanations of the “heart” over “mind” domination seem to be clear (Jarymowicz and Bar-Tal, 2006). We argue (Jarymowicz and Szuster, 2014) that there is a high probability that “mind” will dominate “heart,” but only in individuals who have developed evaluative standards through reflective thinking. In particular an individual has to connect standards based on abstract concepts of good (e.g., *humanitarianism*) and evil (e.g., *violence*) with concrete, real-life issues and applications (e.g., equal rights; discrimination). Empirical correlative studies showed that participants who are able to generate referents of abstract evaluative concepts (like *loyalty*) tend to display relatively little discrimination against out-groups (Jarymowicz, 2012).

## Author contributions

The author confirms being the sole contributor of this work and approved it for publication.

### Conflict of interest statement

The author declares that the research was conducted in the absence of any commercial or financial relationships that could be construed as a potential conflict of interest.
